# Supplementary material for: The multiple antibiotic resistance operon of enteric bacteria controls DNA repair and outer membrane integrity
Source: Nat Commun. 2017 Nov 13;8:1444. doi: 10.1038/s41467-017-01405-7 (PMC5684230; doi:10.1038/s41467-017-01405-7)
Supplement: Supplementary file 1 — Supplementary Information [file 41467_2017_1405_MOESM1_ESM.pdf]

a

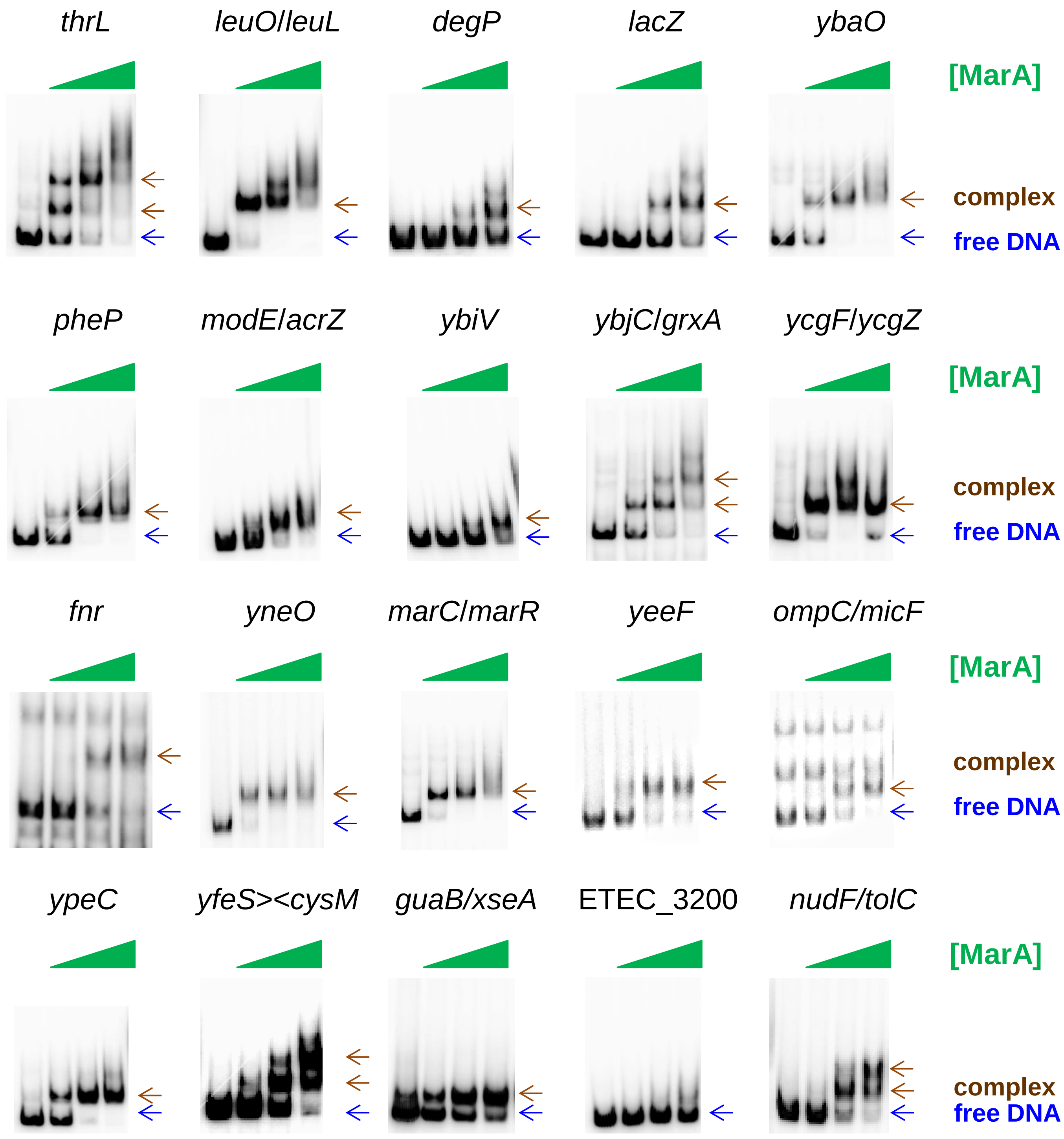
thrL
leuO/leuL
degP
lacZ
ybaO
[MarA]
complex
free DNA
pheP
modE/acrZ
ybiV
ybjC/grxA
ycgF/ycgZ
[MarA]
complex
free DNA
fnr
yneO
marC/marR
yeeF
ompC/micF
[MarA]
complex
free DNA
ypeC
yfeS><cysM
guaB/xseA
ETEC_3200
nudF/tolC
[MarA]
complex
free DNA

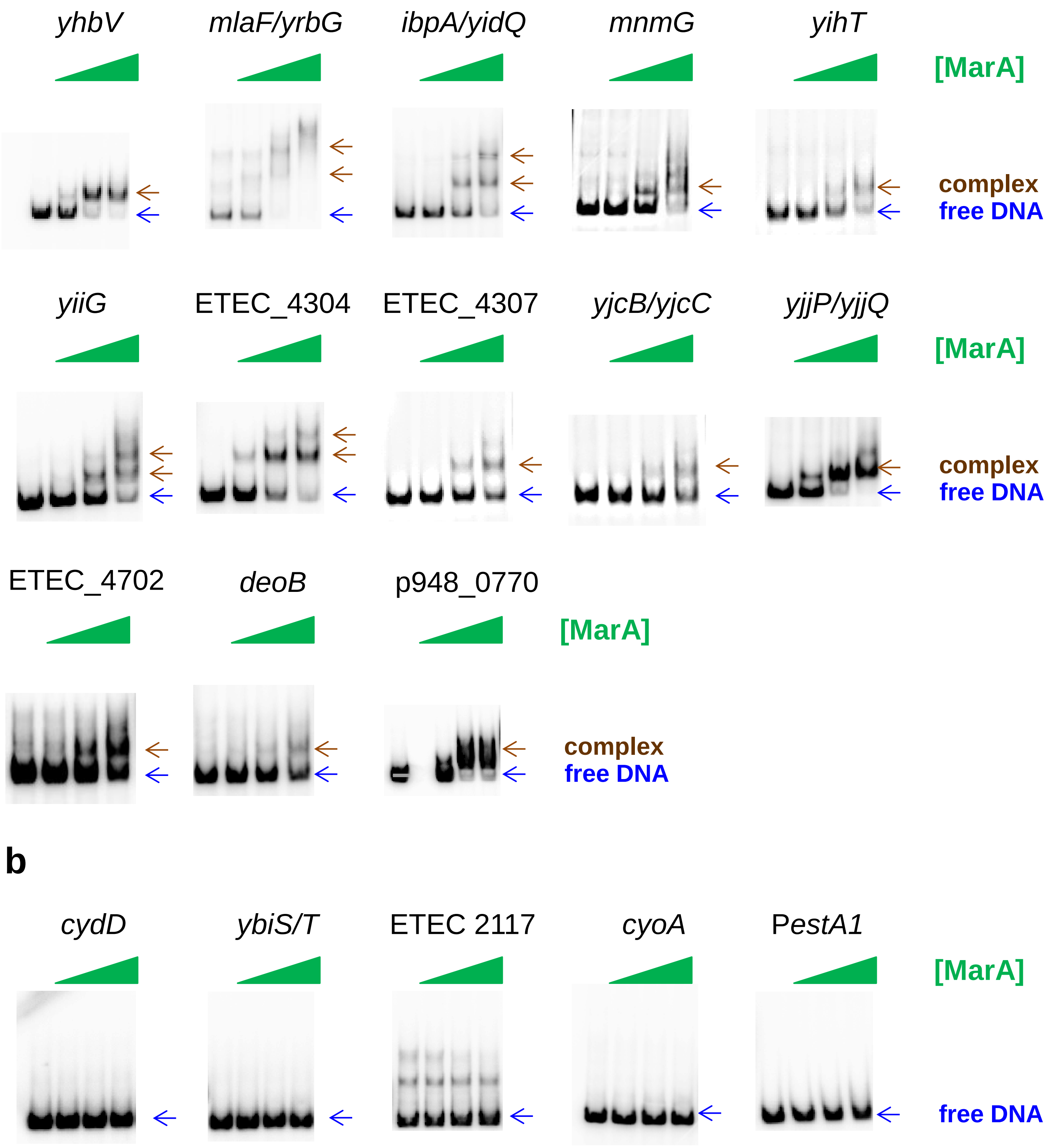

Supplementary Figure 1: Binding of MarA to DNA fragments derived from ChIP-seq binding peaks for MarA. Panel a) illustrates results of electrophoretic mobility shift assays with different DNA fragments containing the marbox. Panel b shows binding of equivalent MarA concentrations to DNA fragments containing no marbox. MarA was added at a final concentration of 0.3, 1.0, and 1.7 μM as indicated by the triangle. The location of free DNA, and DNA bound by MarA, is indicated.

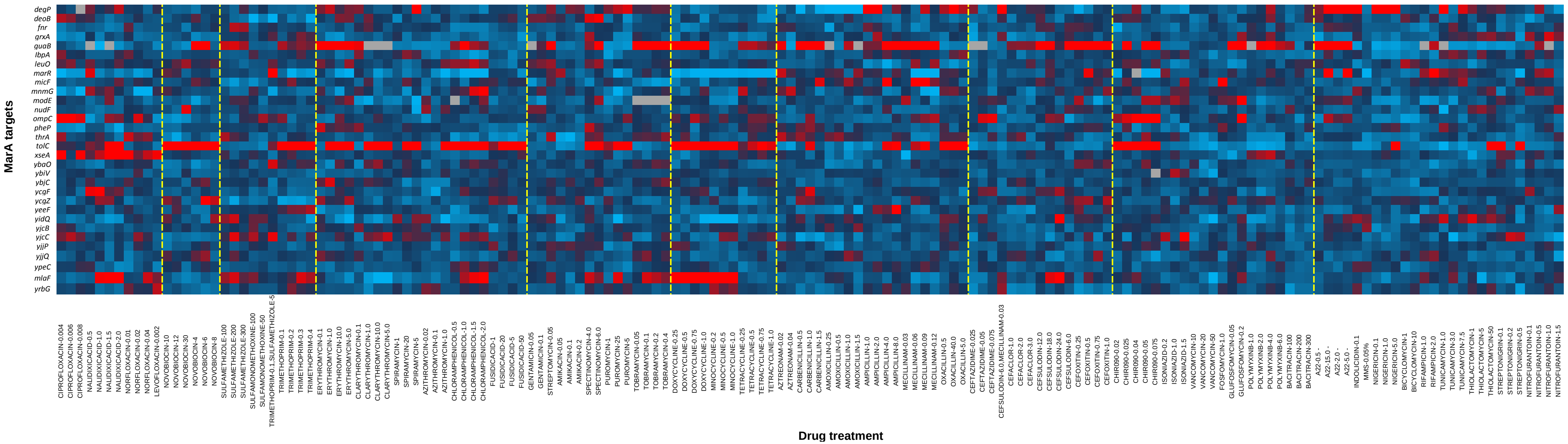

Supplementary Figure 2. Phenotypic landscape of the MarA regulon. The heatmap illustrates fitness scores[29] of strains lacking MarA target genes (y-axis) compared to the wild type parent strain. Red indicates a fitness decrease and pale blue indicates a fitness increase. Strains were grown in the presence of different antibiotics (x-axis). The antibiotics are clustered according to the cellular process targeted. Individual row and column names are provided. See reference 29 for further details.

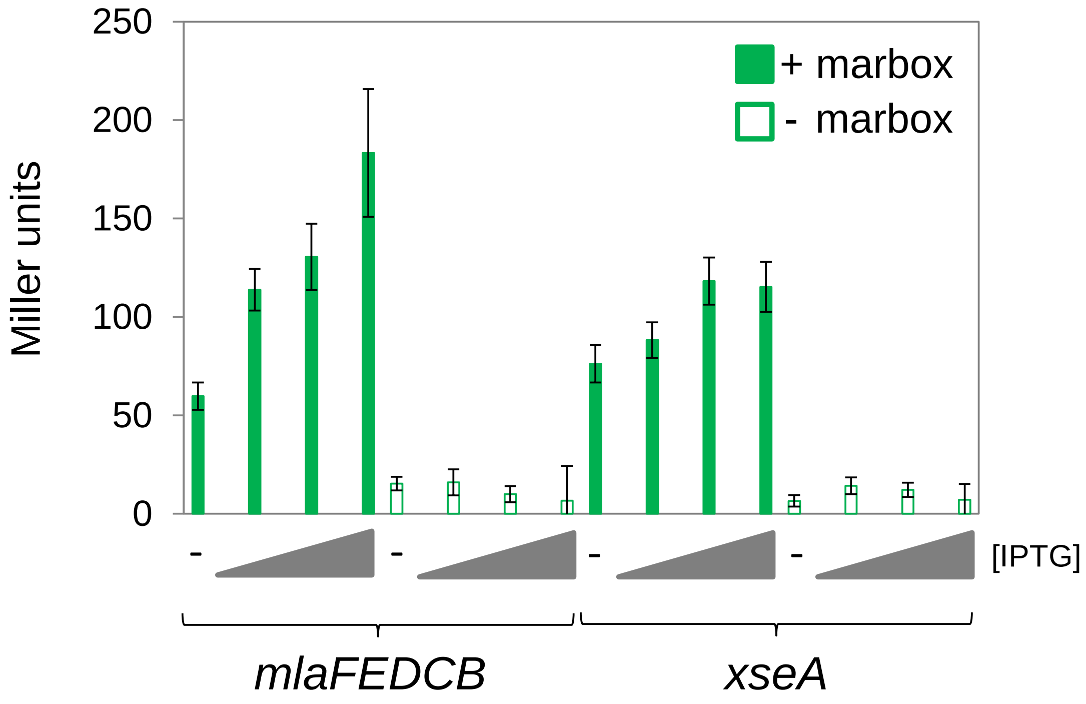

Supplementary Figure 3: Activation of *mlaFEDCB* and *xseA* by increasing intracellular MarA: a requirement for the marbox. The result of a β-galactosidase assay done using lysates of T7 express cells transformed with derivatives of the *lacZ* reporter plasmid, pRW50. Activity values obtained using empty pRW50 vector have been subtracted. Additional MarA is provided by plasmid pET21a*marA* that encodes *marA* under the control of an IPTG inducible promoter. Error bars represent standard deviation (n=3).

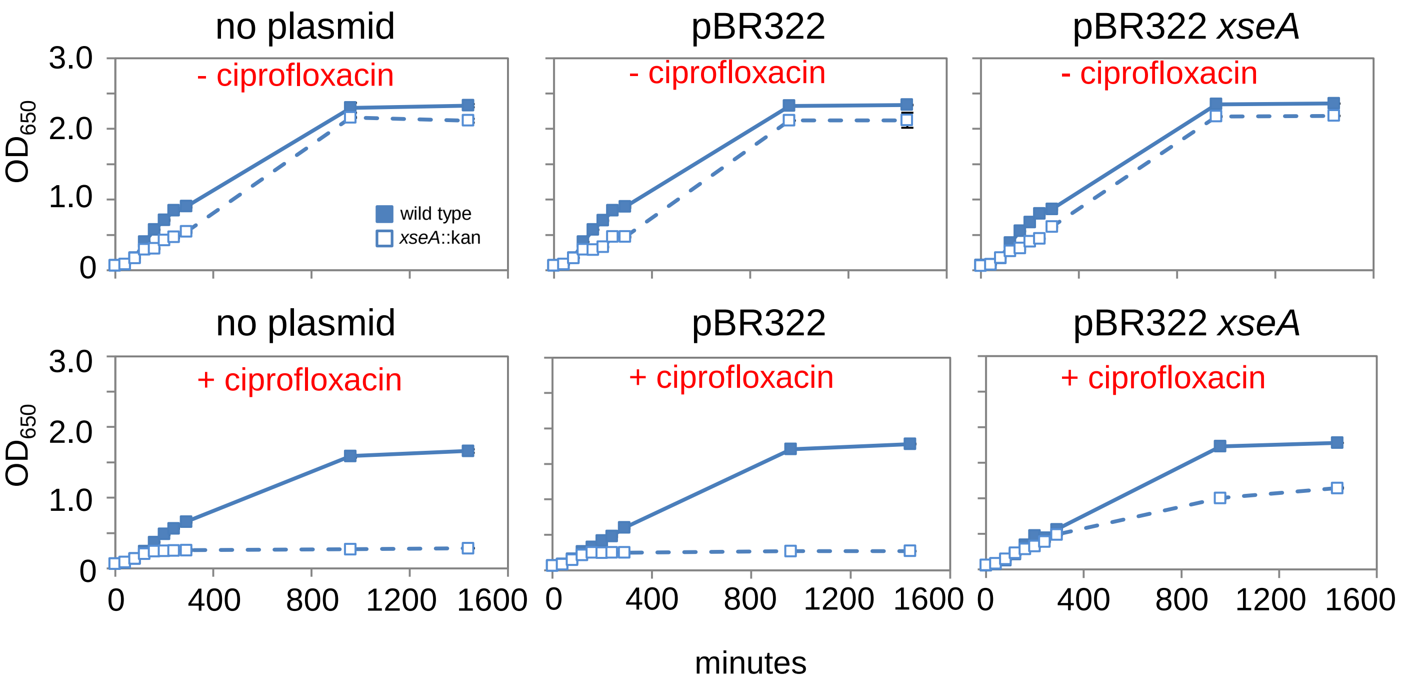

Supplementary Figure 4: Complementation of the *xseA*::kan phenotype by *xseA*. The graph shows OD_{650} values obtained for liquid cultures of strain BW25113 *xseA*::kan grown in the presence or absence of 0.005 µg/ml ciprofloxacin. The BW25113 *xseA*::kan cells were transformed with pBR322 derivatives encoding *xseA* under the control of the *xseA*1 fragment. Error bars represent standard deviation (n=3).

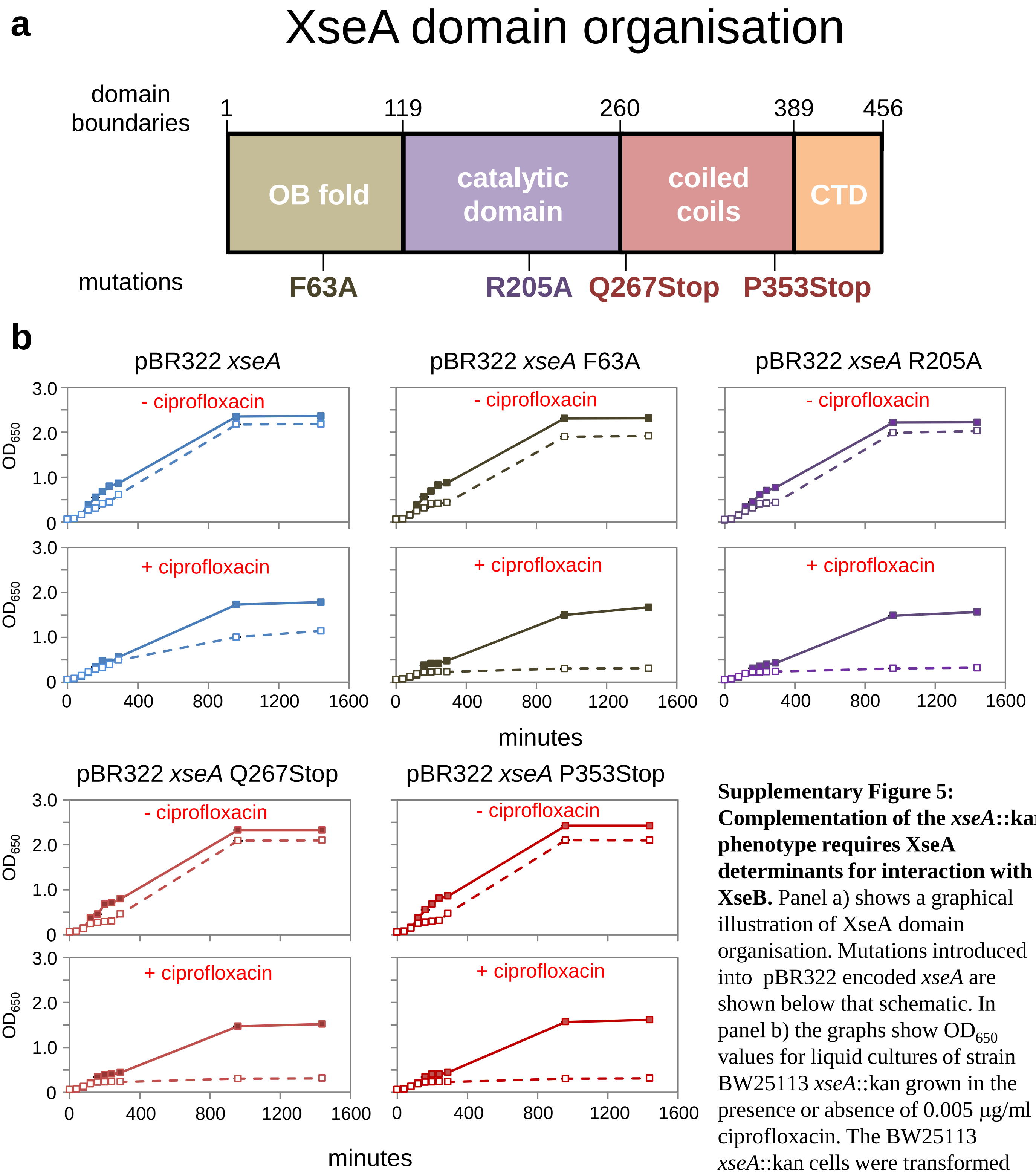

Supplementary Figure 5: Complementation of the *xseA*::kan phenotype requires XseA determinants for interaction with XseB. Panel a) shows a graphical illustration of XseA domain organisation. Mutations introduced into pBR322 encoded *xseA* are shown below that schematic. In panel b) the graphs show OD_{650} values for liquid cultures of strain BW25113 *xseA*::kan grown in the presence or absence of 0.005 µg/ml ciprofloxacin. The BW25113 *xseA*::kan cells were transformed with pBR322 derivatives encoding *xseA* under the control of the *xseA*1 fragment. Error bars represent standard deviation (n=3).

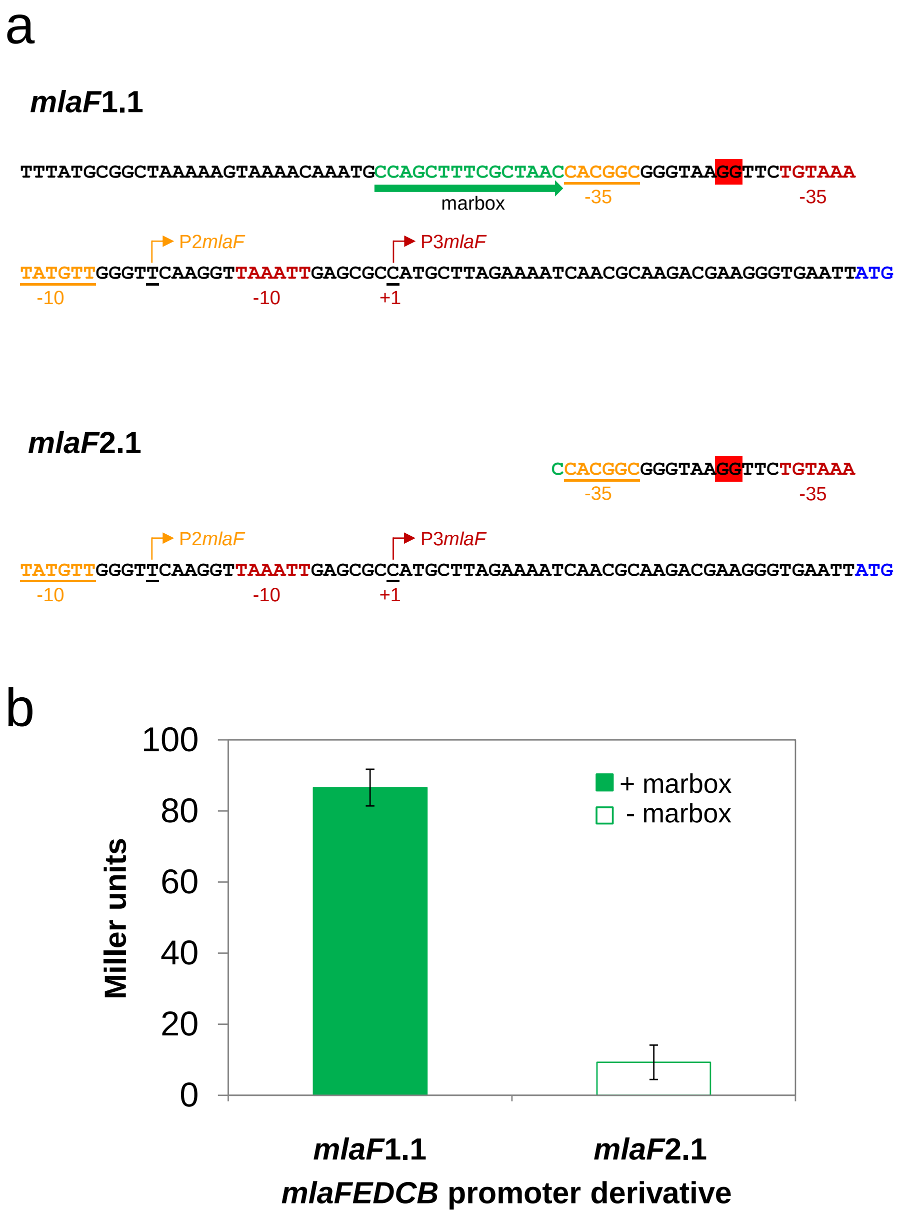

Supplementary Figure 6: Effect of *mlaFEDCB* P1 promoter inactivation. Panel a) shows the DNA sequence of the *mlaF*1.1 and *mlaF*2.1 DNA fragments. The *mlaF* start codon is shown in blue and the marbox is in green. Transcription start sites (+1) are underlined and further highlighted by a bent arrow. The P1 promoter has been inactivated by changing the sequence of the -10 element from 5'-TATTCT-3' to 5'-GGTTCT-3. The associated mutations are highlighted by the red box. Panel b) shows results of β-galactosidase assays using lysates of strain JCB387 transformed with pRW50 carrying either the *mlaF*1.1 or *mlaF*2.1 fragment upstream of *lacZ*. Error bars represent standard deviation (n=3).

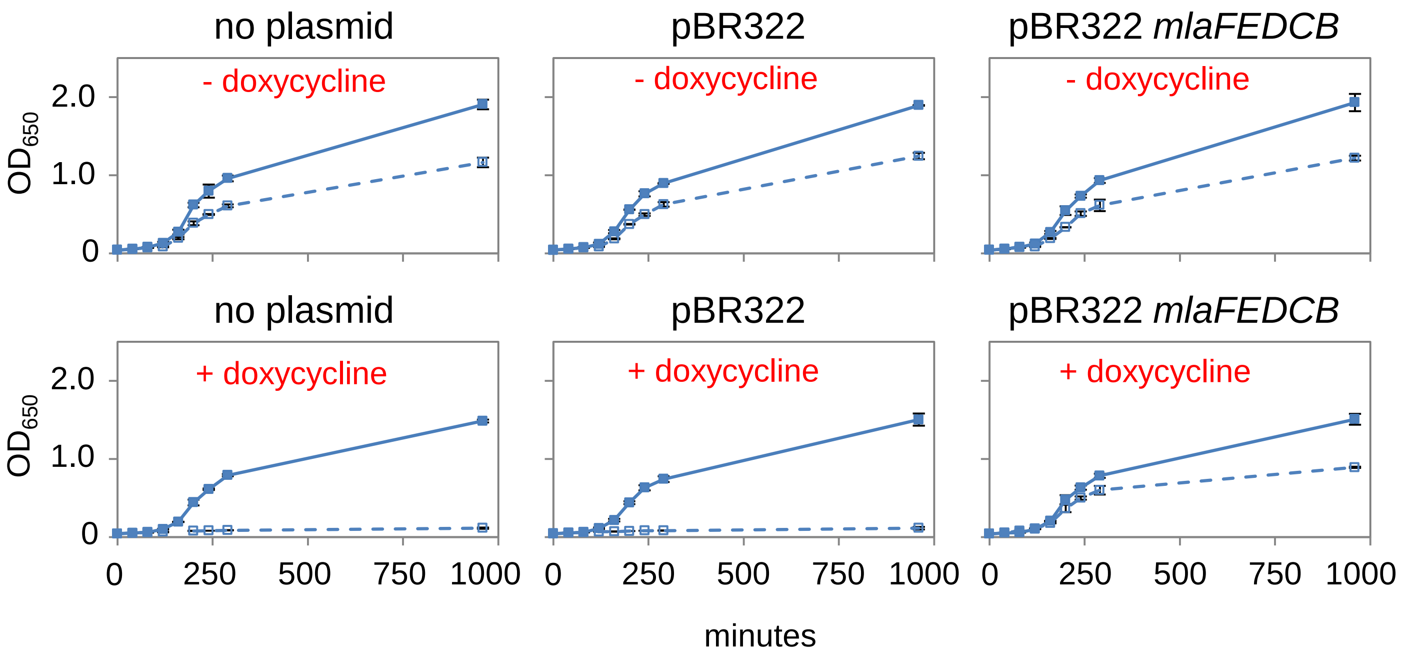

Supplementary Figure 7: Complementation of the *mlaE*::kan phenotype by *mlaFEDCB*. The graph shows OD_{650} values obtained for liquid cultures of strain BW25113 *mlaE*::kan grown in the presence or absence of 1.0 µg/ml doxycycline. The BW25113 *mlaE*::kan cells were transformed with pBR322 derivatives encoding *mlaFEDCB* under the control of the *mlaF*1 fragment. Error bars represent standard deviation (n=3).

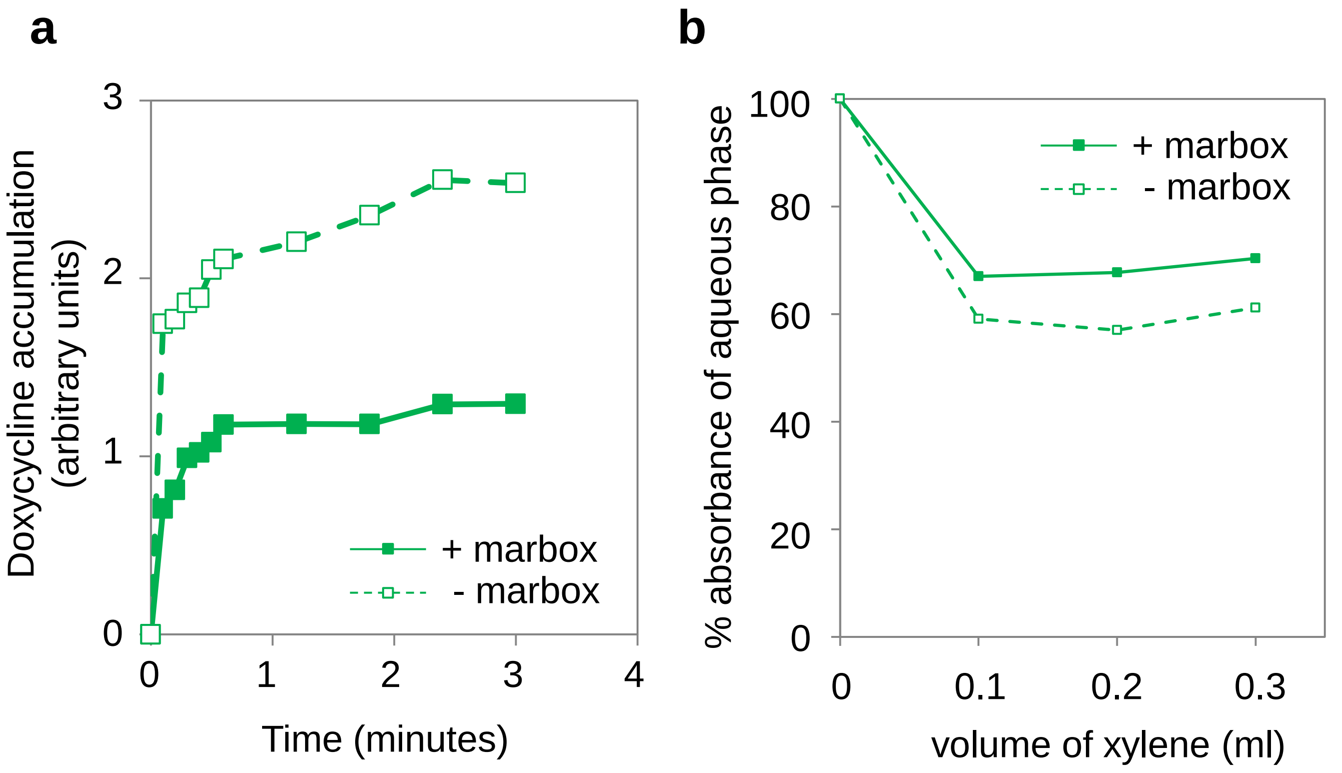

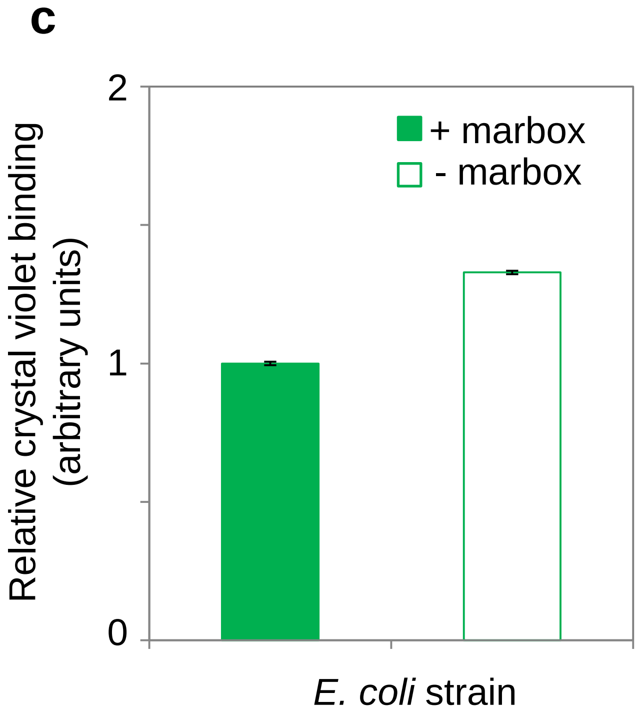

Supplementary Figure 8: The *mlaFEDCB* *marbox is required for* effective barrier function and optimal surface hydrophobicity. Panel a) shows accumulation of doxycycline as a function of time for BW25113 *mlaE*::*kan* cells transformed with pBR322 encoding *mlaFEDCB* under the control of the *mlaF*1 (+marbox, solid line) or the *mlaF*2 (-marbox, dashed line) promoter fragments. Panel b) depicts changes in absorbance of an aqueous suspension of bacterial cells after mixing with p-xylene. The % absorbance is relative to that obtrained with no p-xylene, at equilibrium. Data points are coloured as in panel a). The indicated volume of p-xylene is shown on the x-axis. The bar graph (c) shows relative crystal violet adsorption by BW25113 *mlaE*::*kan* cells transformed with pBR322 encoding *mlaFEDCB* under the control of the *mlaF*1 (solid bar) or the *mlaF*2 (open bar). Error bars represent standard deviation (n=3).

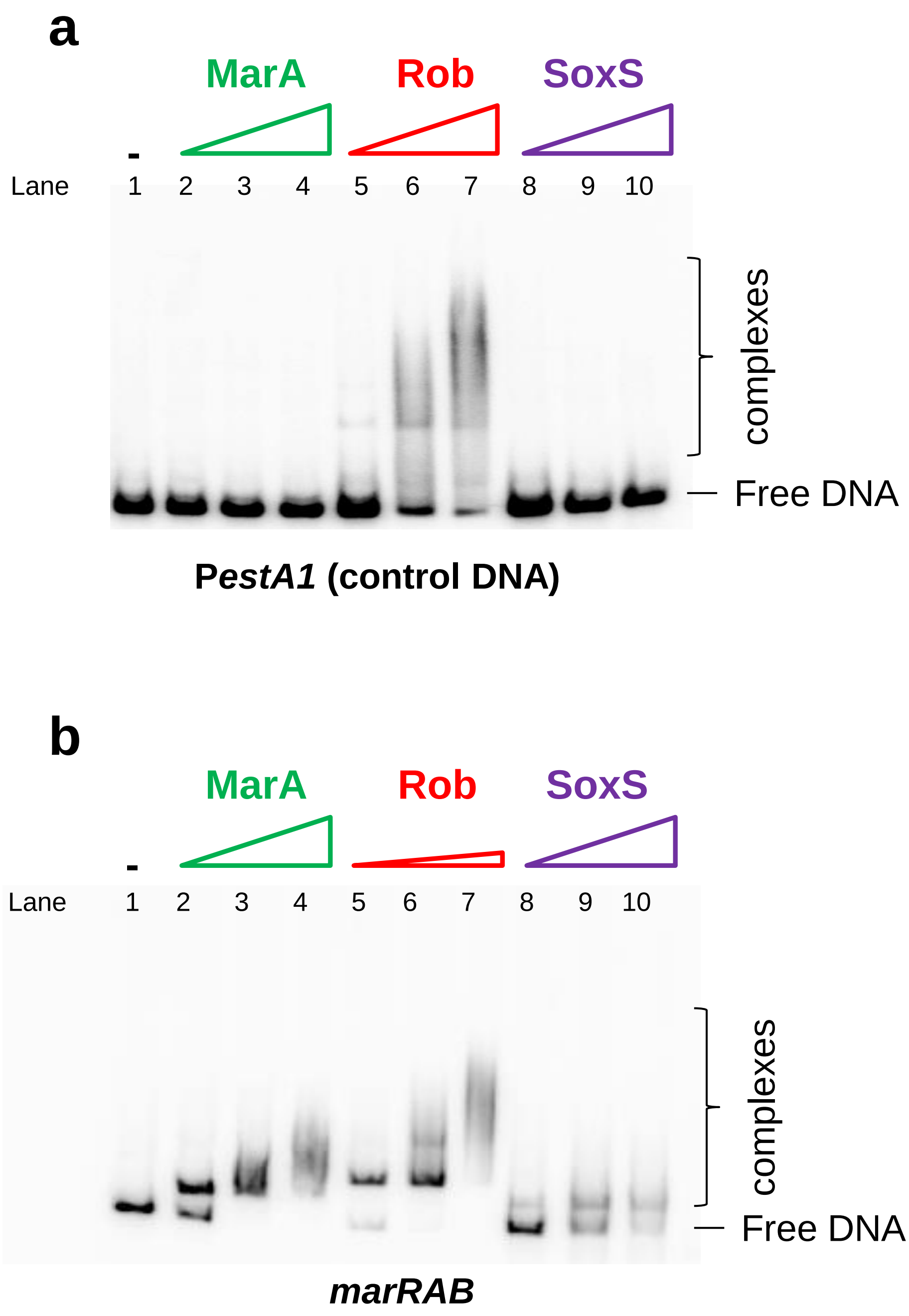

Supplementary Figure 9: Rob binds DNA with high affinity but low specificity. Panel a) shows the results of an electrophoretic mobility shift assay. Proteins (0.4, 1.2 or 2 μM) were incubated with the P*estA*1 DNA fragment that does not contain a marbox. Panel b) shows binding of MarA and SoxS (0.4, 1.2 or 2 μM) or Rob (0.08, 0.24 or 0.4 μM) to a DNA fragment containing the *marRAB* promoter.

a

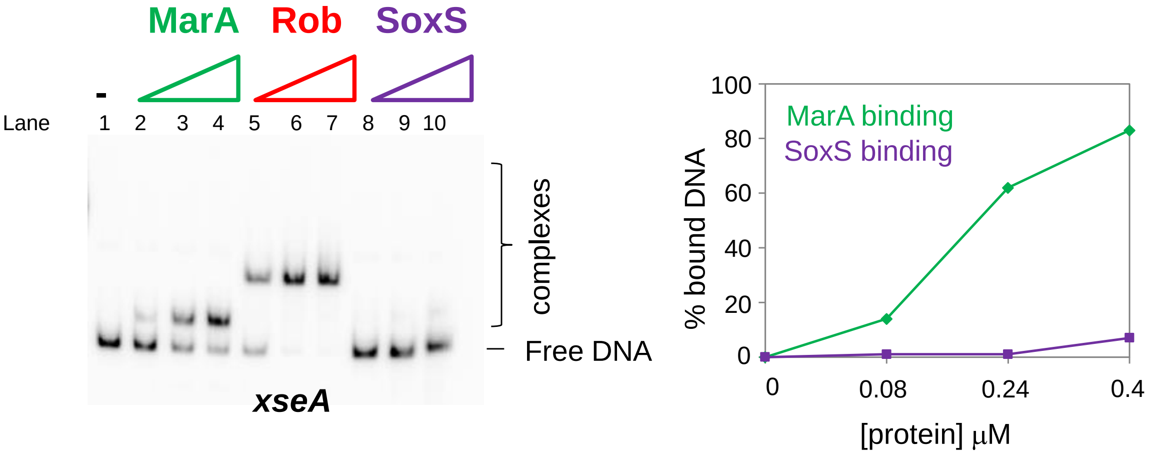

b

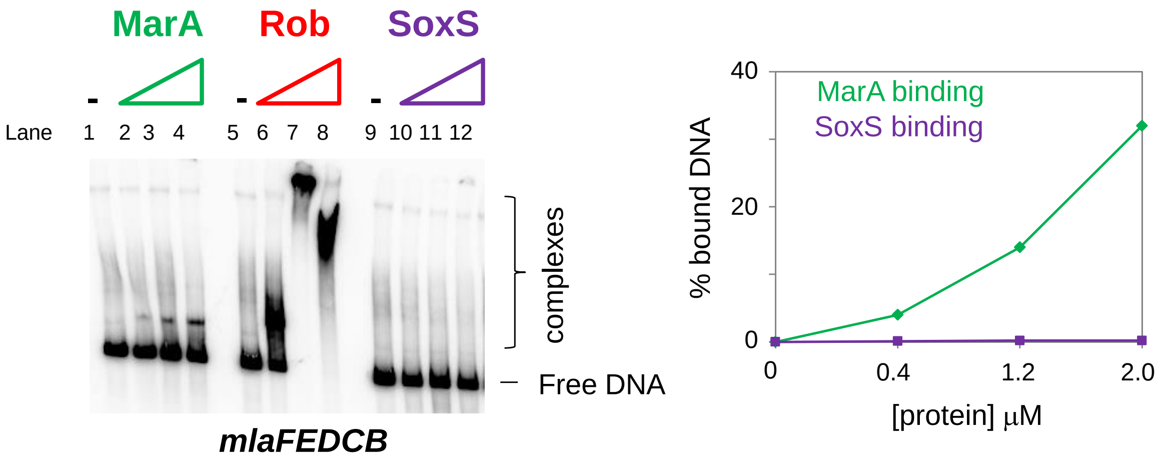

Supplementary Figure 10: The *xseA* and *mlaFEDCB* and regulatory regions preferentially bind MarA rather than SoxS. The figure shows binding of MarA, Rob or SoxS to the a) *xseA*1 or b) *mlaF*1 DNA fragments. For each experiment, the % free DNA was determined for each protein concentration as illustrated in the line graphs.

a

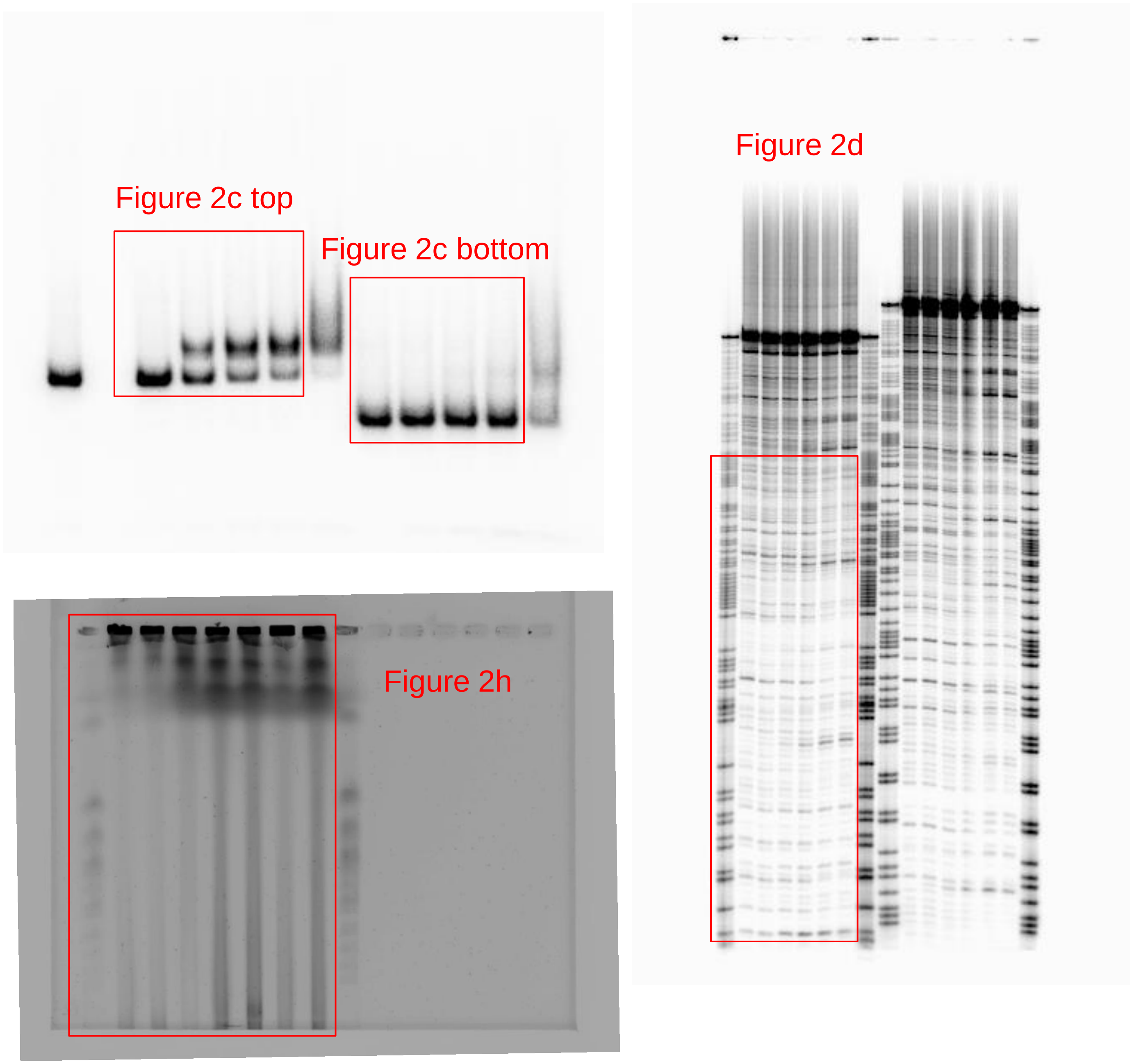

b

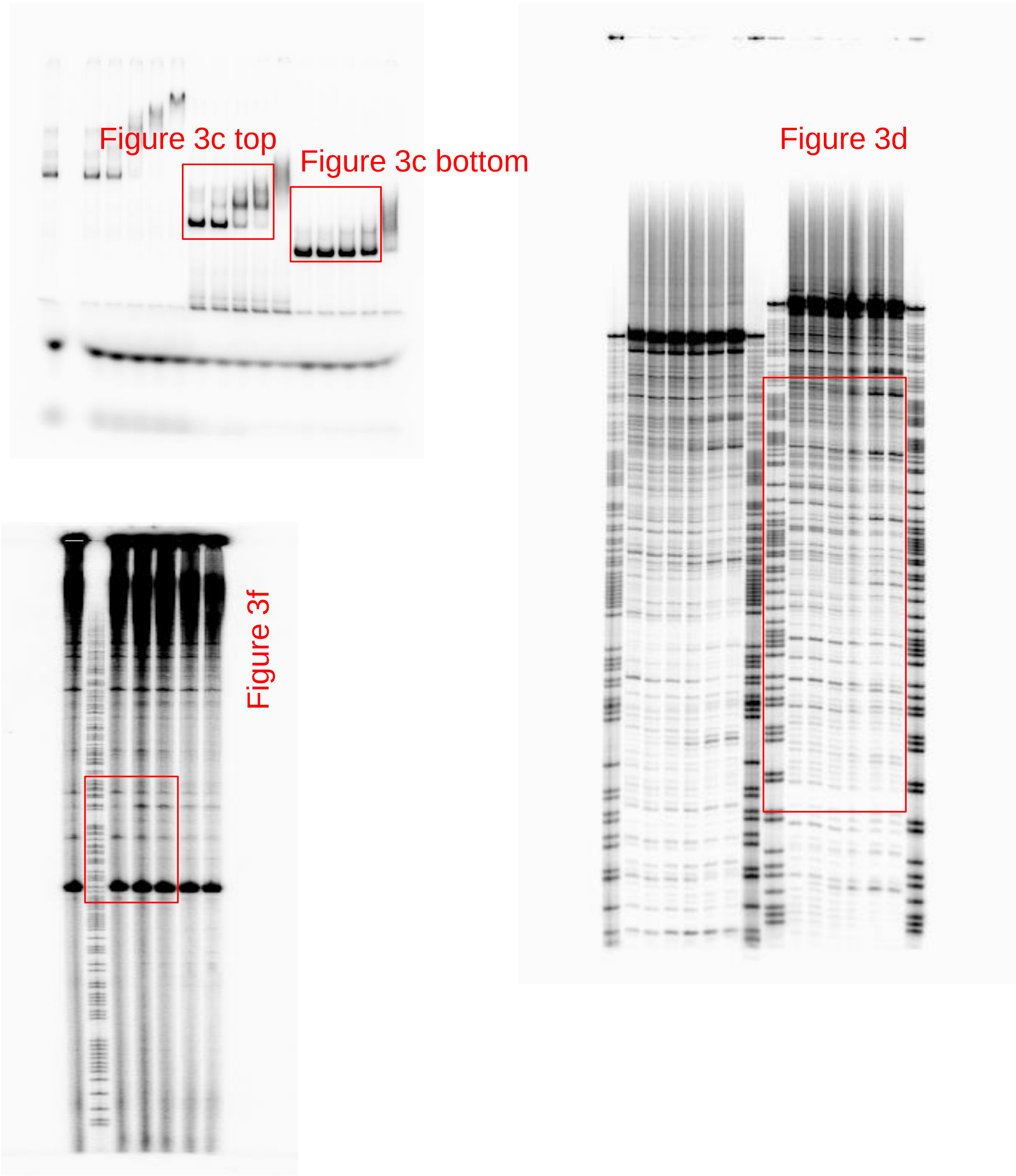

c

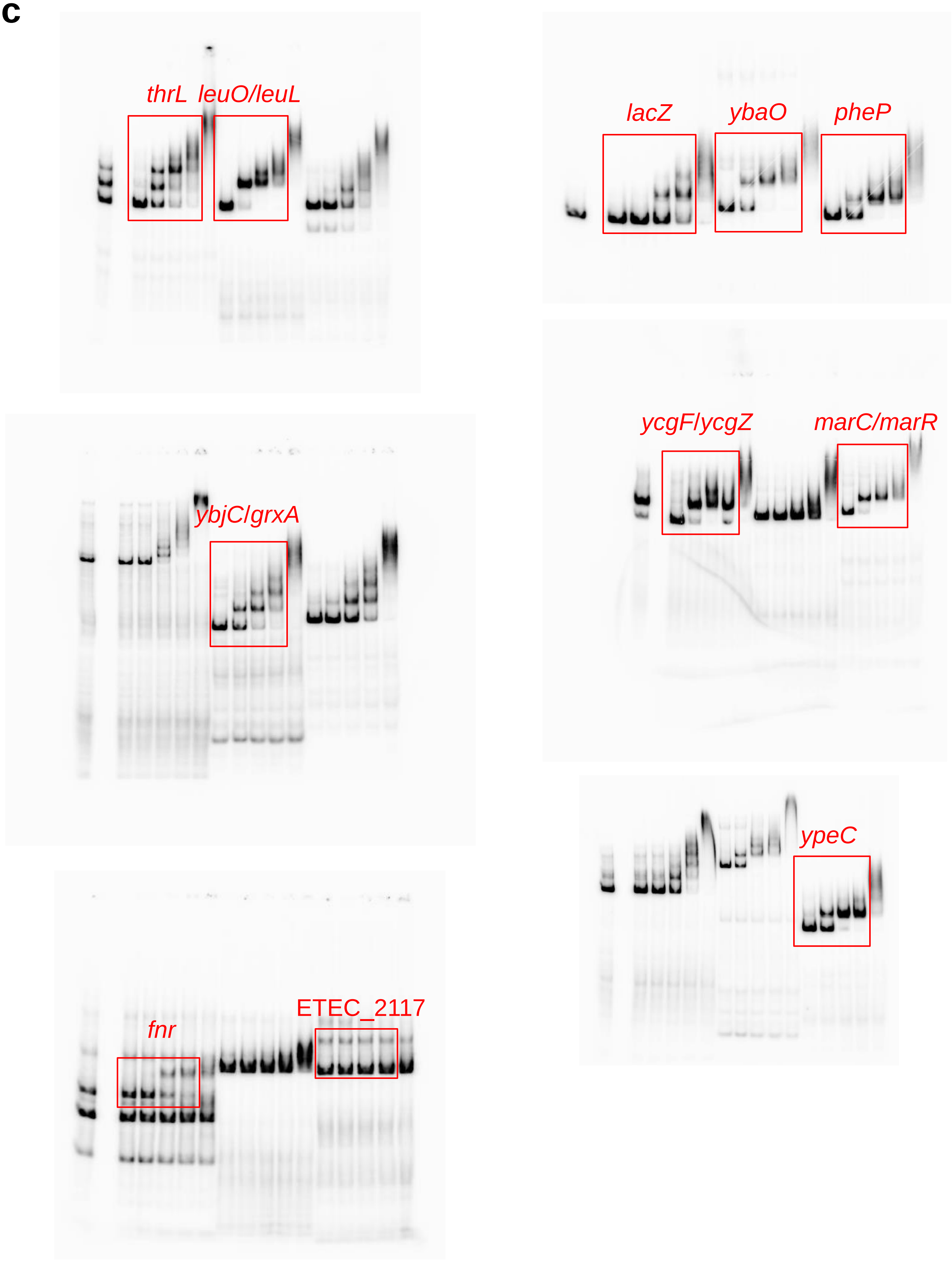
thrL
leuO/leuL
lacZ
ybaO
pheP
ybjC/grxA
ycgF/ycgZ
marC/marR
ypeC
fnr
ETEC_2117

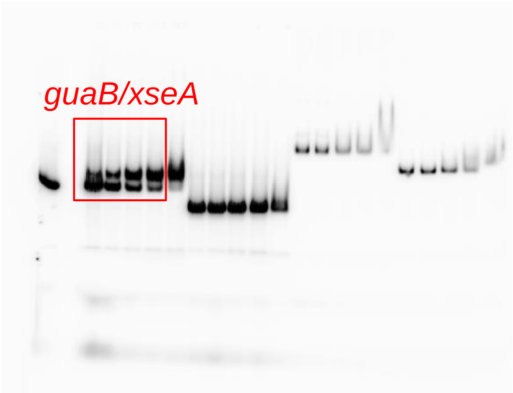
guaB/xseA

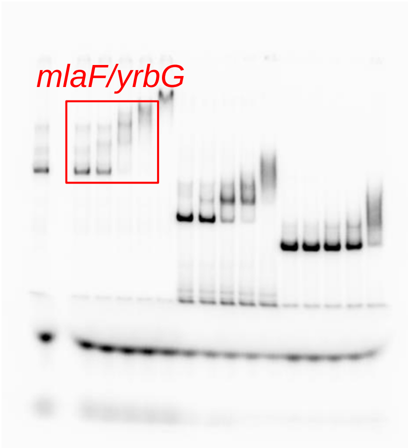
mlaF/yrbG

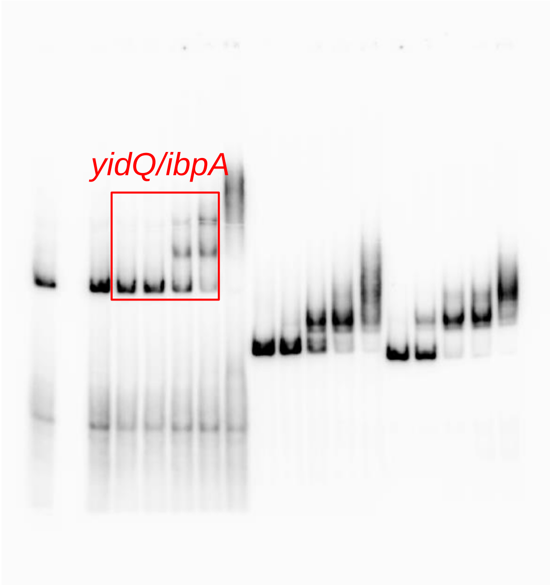
yidQ/ibpA

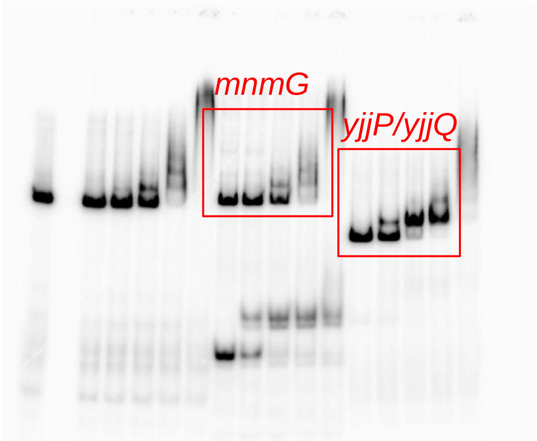
mnmG
yjjP/yjjQ

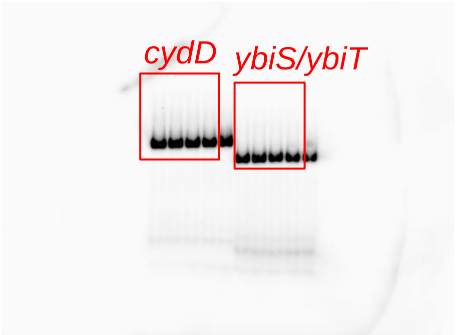
cydD
ybiS/ybiT

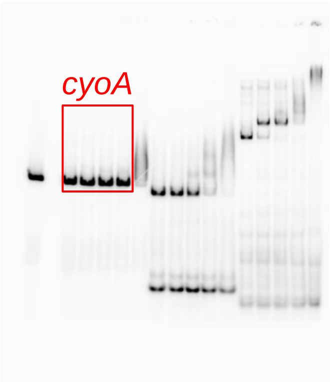
cyoA

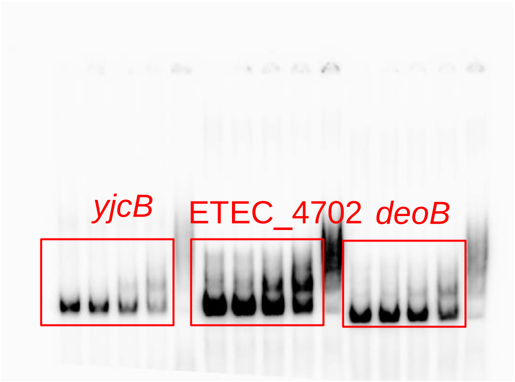
yjcB
ETEC_4702
deoB

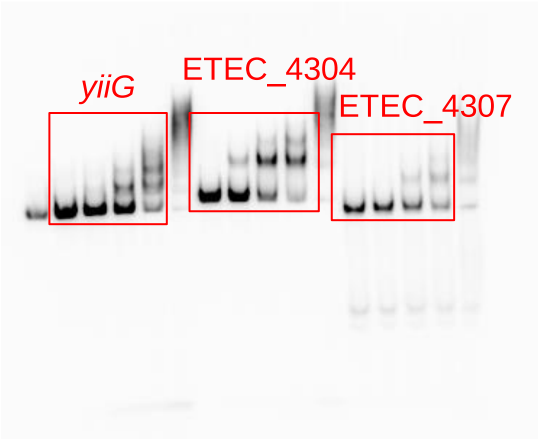
yiiG
ETEC_4304
ETEC_4307

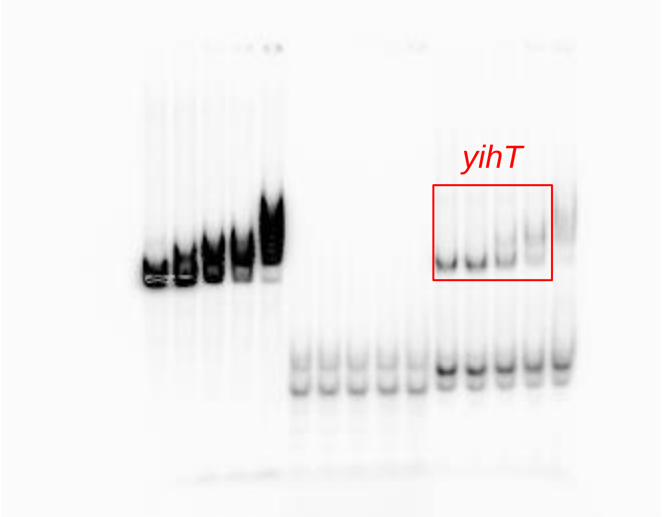
yihT

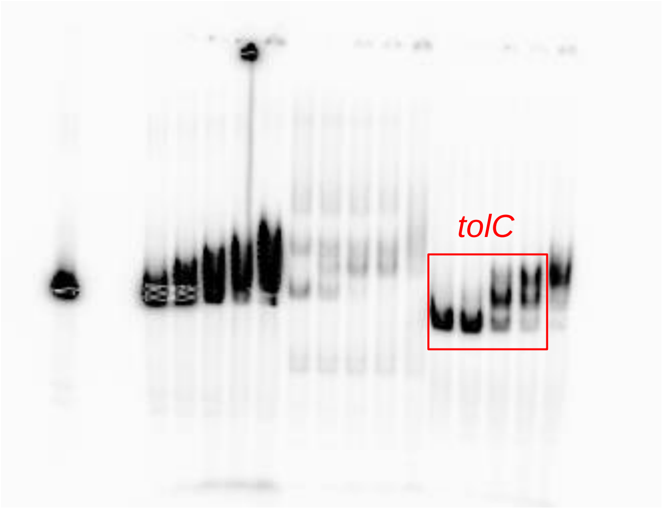
tolC

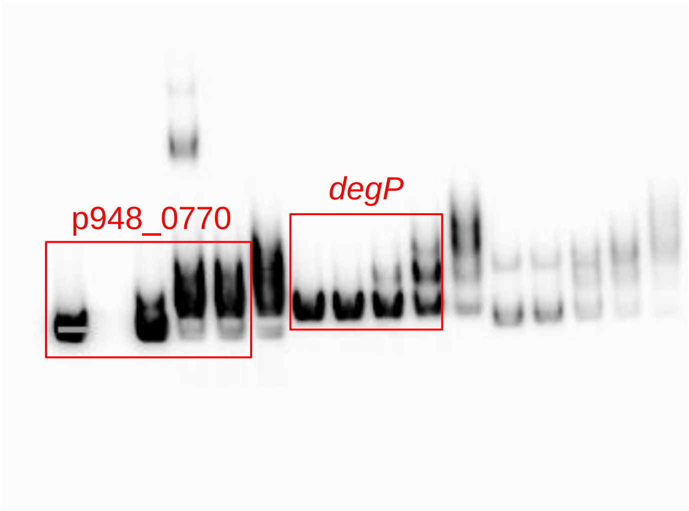
p948_0770
degP

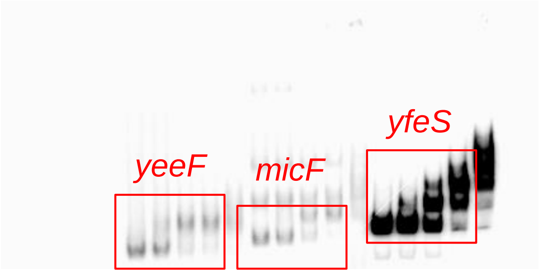
yeeF
micF
yfeS

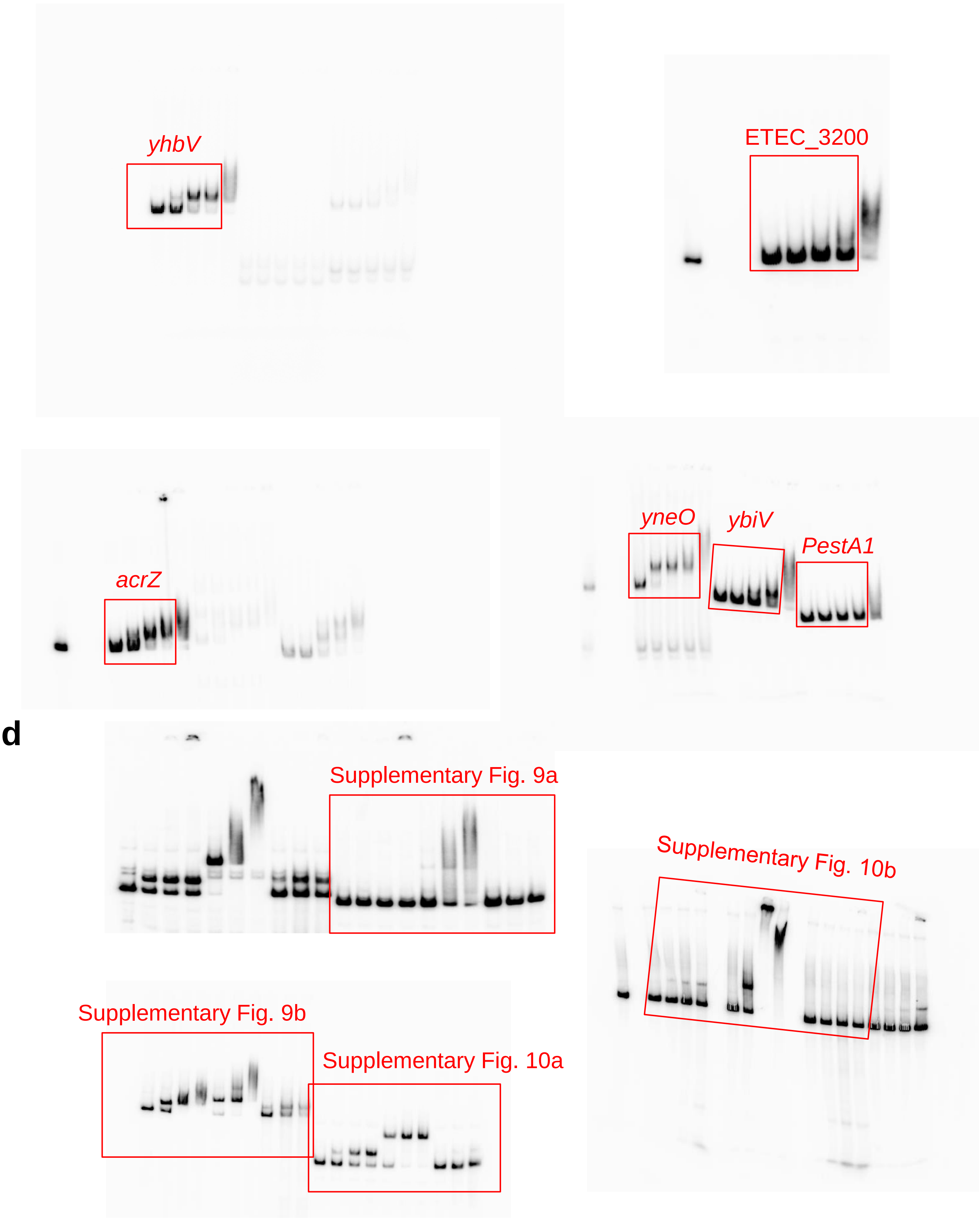

Supplementary Figure 11: Uncropped gel images. The images correspond to gels presented in a) Figure 2 b) Figure 3 c) Supplementary Fig. 1 and d) Supplementary Fig. S9-10.
